# Solvent-Assisted Vapor Condensation: A Strategy to Enhance Bio-Oil Yield and Quality from the Pyrolysis of Agro-Industrial Waste

**DOI:** 10.3390/molecules30193945

**Published:** 2025-10-01

**Authors:** Jelena Isailović, Emilija Vukićević, Jan Schwarzbauer, Steva Lević, Mališa Antić, Ilija Brčeski, Branimir Jovančićević, Vesna Antić

**Affiliations:** 1 Mining Institute Ltd. Belgrade, Batajnički Put 2, 11080 Zemun, Serbia; 2 Faculty of Chemistry, University of Belgrade, Studentski Trg 12-16, 11000 Belgrade, Serbia; emilija@chem.bg.ac.rs (E.V.); ibrceski@chem.bg.ac.rs (I.B.); bjovanci@chem.bg.ac.rs (B.J.); 3 Institute for Organic Biogeochemistry in Geo-Systems, RWTH Aachen University, Lochnerstr. 4-20, 52056 Aachen, Germany; jan.schwarzbauer@emr.rwth-aachen.de; 4 Faculty of Agriculture, University of Belgrade, Nemanjina 6, 11080 Zemun, Serbia; slevic@agrif.bg.ac.rs (S.L.); mantic@agrif.bg.ac.rs (M.A.); vantic@agrif.bg.ac.rs (V.A.)

**Keywords:** pyrolysis, waste biomass, bio-oil, light and heavy liquid fractions, vapor trapping, acetone

## Abstract

The paper presents the effect of an organic solvent on the efficiency of vapor condensation from pyrolysis processes applied to agricultural waste, with the intention of optimizing the trapping procedure for more volatile components. Therefore, the effect of the use of acetone in the vapor trapping system on the yield and composition of liquid fractions (bio-oils) obtained from the pyrolysis of selected agricultural waste, including corn, tomato, and tobacco, was investigated. The focus was placed on evaluating how solvents influence the quality, yield, and composition of bio-oil, as well as whether they are necessary in the pyrolysis process. Acetone, a polar solvent with low human toxicity and the possibility of regeneration after pyrolysis, was selected for bio-oil condensation due to its effectiveness in dissolving polar compounds formed during the pyrolysis of lignocellulosic biomass. Pyrolysis was conducted at 400 and 500 °C for 30 min, to collect light and heavy fractions, which were subsequently analyzed to assess acetone’s influence. The results showed that acetone positively affected corn bio-oil yield (from 44.57% without acetone to 52.13% with acetone) and improved quality by reducing moisture (from 61.82% to 12.83%) and oxygen content (from 86.50% to 47.10%). An increase in calorific value was also observed in both corn varieties, while the effect was minimal in tobacco and nearly negligible in tomato. The obtained parameter values indicated that satisfactory results can also be achieved without the use of a solvent, representing a step toward simplified pyrolysis. GC-MS analysis confirmed that phenols and their derivatives were the dominant compounds, while FTIR analysis verified the presence of functional groups of the identified compounds. Increasing the temperature generally increased both the yield and calorific value of most samples. Light and heavy fractions were separated during condensation to improve collection efficiency and enable better quality control. Although this step adds complexity and potential contamination risks, it allows more effective utilization of the fractions. These results provide a valuable foundation for optimizing the valorization of agricultural waste through pyrolysis-based biofuel production.

## 1. Introduction

Due to the increasing consumption of fossil fuels, which are becoming increasingly scarce while the demand for them is growing, scientists face various challenges in searching for alternative solutions [1]. The current challenge is to explore the potential of renewable energy sources, such as biomass, to obtain suitable replacements for fossil fuels, ideal for use in internal combustion engines [2]. Such sustainable fuels obtained from biomass would also significantly reduce greenhouse gas emissions and, consequently, lead to climate change mitigation. An essential issue in the search for sustainable replacements for fossil fuels is the conditions under which they are obtained and their implications for the quality of the resulting products. One of the potential replacements for fossil fuels is biofuels, which can be produced by various thermal processes, especially pyrolysis [3]. While the approach of using different solvents in the pyrolysis process has been previously studied, a direct and systematic comparison between solvent-free and solvent-assisted pyrolysis, especially with acetone as the selected solvent, has not been adequately addressed. The pyrolysis process typically requires a carrier gas and a solvent to separate the produced liquid from the solid and gaseous fractions. Previous studies have examined the effect of different organic solvents on the separation and dissolution of liquid fractions obtained from pyrolysis, i.e., bio-oil samples. Solvents such as chloroform, petroleum ether [4], hexadecane, octane [5], toluene [6], hexane [7], and dichloromethane [8] were commonly used, with the focus placed on the extraction of bio-oils by solvents rather than on the direct collection of condensates in the solvent. This approach generally requires substantial amounts of organic, non-environmentally friendly, solvents to achieve high extraction efficiency, raising questions about both economic feasibility and environmental sustainability. Acetone, a solvent characterized by high volatility, easy regeneration, and affordability [9], was selected for this study not only due to its classification as an environmentally friendly solvent with low toxicity and favorable properties compared to other organic solvents [10], but also because it was identified by Harman-Ware and Ferrell III (2018) as the most effective solvent for bio-oil extraction [11]. Selected as the most suitable solvent for the pyrolysis process due to its ability to retain a larger number of compounds, acetone was used to provide a clearer understanding of the differences between the liquid fractions obtained with and without solvent use. This difference was expected to be clearly observable, and the aim of comparing the results was to support the development of a more environmentally friendly approach in which the use of solvents in pyrolysis could be avoided.

In this study, liquid pyrolysis products obtained from tomato, tobacco, and corn stalks were investigated, as representatives of major agricultural and horticultural crops worldwide that generate significant amounts of residual biomass. The disposal of such residues often involves dumping at legal or illegal landfills or open-field burning in less developed countries such as Serbia, causing environmental damage and contributing to greenhouse gas emissions [12,13]. As a sustainable solution, due to the large amounts of generated residues, the conversion of biomass into energy or value-added products through pyrolysis has been proposed [13].

In more detail, in this work the liquid fraction was obtained by pyrolysis in a tubular furnace, using nitrogen as the carrier gas, at 400 and 500 °C, with and without acetone in the condensation trap. Two fractions were collected: the light fraction, containing water, hydrocarbons, and aromatic compounds with lower boiling points, and the heavy fraction, remaining in the tube, consisting of higher molecular weight compounds with higher boiling points. The separation of these bio-oil fractions may facilitate product handling by reducing the number of purification steps. The bio-oil was characterized by physicochemical parameters (pH, moisture content, ash content, and calorific value) and by elemental composition to evaluate the influence of acetone and possible environmental impacts, including the presence of toxic elements (Pb, Cd, As, Cr, Hg, Cu). In addition, the contents of total S, Si, Ca, K, P, and Mg were determined, as they are related to the amount of ash remaining after combustion. For insights into the molecular composition, FTIR analysis was performed and compared with the GC-MS results to provide a more detailed characterization of the bio-oil. This study compared the advantages and disadvantages of the pyrolysis process without the use of solvents and application of acetone as an efficient solvent for polar compounds in bio-oil condensation and separation, focusing on its impact on the physicochemical properties.

## 2. Results and Discussion

### 2.1. The Yield of Bio-Oil Fractions

**Figure 1:**
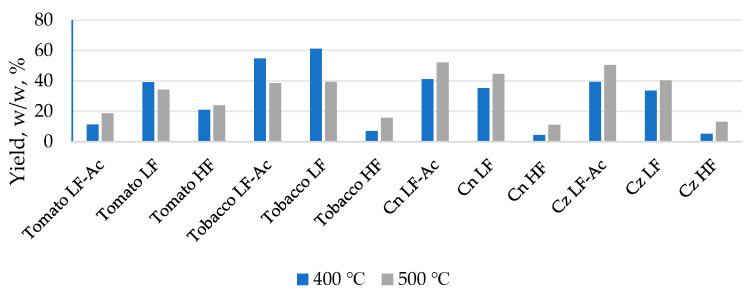
Yield of obtained liquid fractions.

Initial TGA revealed a maximum degradation rate of the used waste biomass of around 400 °C. Therefore, pyrolysis was initially performed at 400 °C, and then experiments were conducted at 500 °C to investigate whether temperature affects the yield of the liquid fraction, as described in the literature [14]. After pyrolysis at two different temperatures, the liquid products collected with and without acetone were characterized. The yield of liquid fractions was calculated and shown in Figure 1. The results show that the highest yield of light fraction, condensed without the acetone, was obtained from tomato (34.19%) and tobacco (39.37%) biomass. In contrast, regarding the corn biomass, the highest yield was for the light fraction condensed in the presence of acetone (52.13% for Cn and 50.44% for Cz). Also, it was noticed that the yield of light fractions condensed without acetone for both types of corn is significant and higher compared to tomato and tobacco (44.57% for Cn and 40.37% for Cz). The heavy fractions obtained in the experiments with and without acetone were combined into a single heavy fraction sample. This was performed to obtain a more significant sample amount since the proportion of the individual heavy fraction was small. This approach provides sufficient material for all necessary analyses and was adopted because GC-MS analysis revealed minimal differences in the composition of the single heavy liquid fractions. GC-MS analysis also confirmed the absence of acetone after evaporation. The yields of heavy fractions are significantly lower for all samples, with the lowest being in corn Cn (11.03%) and Cz (13.13%), slightly higher in tobacco (15.76%), and the highest in tomato (23.91%). The composition of the initial biomass can explain these results. Corn biomass has a lower lignin content, which is known to be inversely proportional to the expected liquid fraction yield [15].

It is obvious that the application of acetone increased the proportion of liquid fractions for both types of corn, while it had the opposite effect for tomato and tobacco. This result indicates that acetone can dissolve and “retain” readily volatile compounds, which are formed during the pyrolysis process of corn biomass and which, in its absence, are removed by the nitrogen stream passing through the system. In the case of tomato and tobacco, whose structural composition differs from that of maize, the removal of acetone may result in the loss of light compounds, which means that acetone does not retain them effectively. It can be observed that a higher proportion of the liquid fraction corresponds to samples with a greater number of different compounds, among which those with higher molecular mass and boiling points dominate, but the number of identified compounds is not proportional to the proportion of the liquid fraction. However, tomato HF and corn LF samples deviate from this trend. A possible explanation is that tomato HF may contain more thermally labile compounds that undergo secondary degradation or escape as gases at higher temperatures, while in corn LF the fraction separation and solvent effect might have altered the distribution of volatiles. In addition, the higher temperature influenced the increase in the proportion of the liquid fraction in most samples, which was expected for lignocellulosic biomass samples. However, in the samples labeled as “tomato light fraction without acetone” and “tobacco light fraction with and without acetone,” the increase in temperature had the opposite effect, i.e., with increasing temperature, the proportion of the liquid fraction decreased. By comparing the two different types of corn, it can be concluded that the proportions of all liquid fractions are almost identical. This is a consequence of the similar lignocellulosic composition, which, in addition to the pyrolysis temperature, has a decisive influence on the proportion of liquid fractions. By comparing the polymeric structure and thermal stability of biomass components, it can be observed that hemicellulose decomposes within the range of 220–315 °C, while cellulose, which is more stable due to its crystalline structure, decomposes at higher temperatures (315–400 °C). Lignin, with its amorphous structure, decomposes over a much wider temperature interval (160–900 °C) [16,17,18]. The decomposition of cellulose and hemicellulose predominantly yields acids, furans, and light carbonyl compounds [19], whereas the pyrolysis of lignin mainly produces phenolic and guaiacol-type compounds [20]. Considering that both corn types contain similar proportions of cellulose, hemicellulose, and lignin, it can be concluded that their polymeric composition governs the formation of comparable amounts of liquid fractions during pyrolysis. Differences in the final chemical profile of the liquids are largely the result of variations in secondary reactions occurring during polymer decomposition, while the overall yield of the liquid phase remains relatively constant due to the structural similarity of the lignocellulosic matrix.

### 2.2. Physico-Chemical Characterization of Bio-Oil Fractions

The pH values were lower in all samples obtained at lower temperatures (Table 1). This indicates that more basic products are formed at higher temperatures, which increases pH values. The presence of acetone increased the pH value of the light fraction of tobacco and both types of corn, while it decreased the pH value in the tomato sample. This change can be attributed to its ability to dissolve and retain the dominant acidic or basic compounds formed during pyrolysis, thereby increasing their extraction; consequently, the pH value becomes either more acidic or more basic, since the degree of hydrolysis depends on the extracted substances. A low pH value has a corrosive effect, which is not desirable for bio-oils [21]. Therefore, an increase in the pH value of bio-oils can be achieved by adding acetone to the trap, where the liquid fraction is condensed and retained. By comparing three different liquid fractions for each type of biomass (LF, LF-Ac, and HF), it can be concluded that the highest pH value is shown by the heavy fraction of tomato, obtained at 500 °C, which is 6.06 (Tomato, HF). For tobacco, the highest value was 4.99 at 500 °C (Tobacco, LF-Ac). For both types of corn, higher values were observed in the light fraction with acetone, ranging from 3.88 at 400 °C (Cz, LF-Ac) to 4.08 at 500 °C (Cn, LF-Ac). The pH values for the heavy fraction were slightly lower, ranging from 3.42 at 400 °C (Cn, HF) to 3.95 at 500 °C (Cz, HF).

Specific properties such as moisture content in pyrolysates result in adverse effects related to their application, such as corrosion or reduced energy efficiency [22]. The moisture content of samples collected with acetone is significantly lower (5.78–20.20% at 400 °C) compared to samples without acetone (51.69–79.45% at 400 °C). Water and bio-oil form an emulsion from which water is challenging to evaporate upon heating. On the other hand, acetone and water are miscible but do not form an azeotropic mixture [23], and their separation requires special equipment. As a result, after acetone evaporation, a certain amount of water remains in the bio-oil, as can be seen from the results obtained in this work. However, acetone and water can form azeotropic mixtures with other compounds, leading to the evaporation of these compounds together with acetone/water. This feature can be considered a drawback of the applied procedure. It is evident that in the pyrolysis of a particular biomass, the water content is lower in the light fraction with acetone and the heavy fraction. As mentioned, the heavy fraction was washed out of the tube with acetone, and then the acetone evaporated. This procedure may indicate that the acetone also affected the moisture content in HF. Also, a higher water content was mainly observed in samples obtained by pyrolysis at a higher temperature. The ash content of all liquid fractions was significantly lower than 5%, which is recommended if the goal is to process bio-oil into an alternative fuel [24].

The calorific value reflects the potential of bio-oil as a fuel. Table 1 shows the calorific value of the bio-oil fractions obtained without and with acetone. In the liquid fractions obtained by pyrolysis of tomato waste biomass, the calorific values of LF-Ac were 26.60 and 27.49 MJ/kg (400 and 500 °C, respectively), while the values of LF were 27.49 and 26.34 MJ/kg (400 and 500 °C, respectively). However, the heavy fraction showed slightly lower calorific values of 24.96 and 26.22 MJ/kg (400 and 500 °C, respectively). For tobacco, samples treated with acetone show slightly higher calorific values, 20.22 and 21.07 MJ/kg, compared to those without acetone, 19.44 and 14.70 MJ/kg. The heavy fraction shows values of 15.21 and 23.22 MJ/kg. Regarding the fractions obtained by pyrolysis of corn Cn, the LF-Ac fraction (500 °C) shows a calorific value of 43.60 MJ/kg, which is almost double the value of LF-Ac at 400 °C, which was 24.85 MJ/kg. The light fraction without acetone showed lower calorific values of 16.05 and 17.93 MJ/kg (400 and 500 °C), while the heavy fraction showed higher calorific values of 26.07 and 27.23 MJ/kg (400 and 500 °C). A similar trend was observed for another type of corn (Cz). At a higher temperature of 500 °C, the calorific value of corn Cz LF-Ac was 26.63 MJ/kg, compared to 22.70 MJ/kg at 400 °C. The light fraction without acetone showed slightly lower values of 21.98 and 23.67 MJ/kg (400 and 500 °C). The heavy fraction showed significantly higher calorific values of 27.64 and 27.60 MJ/kg, comparable to the heavy fraction of corn Cn. The presence of acetone during the condensation of the liquid fraction affects the calorific value to some extent, probably by retaining components in the bio-oil that contribute to the increase in calorific value. Acetone increases the carbon content by extracting and concentrating components with higher calorific value, and it is therefore to be expected that the remaining substances will also exhibit a higher calorific value. Also, acetone can influence the calorific value by reducing the O/C ratio by removing moisture, which increases the calorific value [25]. The O/C ratio is lower in the fractions obtained with acetone compared to those without acetone, which consequently leads to the release of more energy. In addition, when comparing the calorific values of the samples obtained at 400 and 500 °C, it is evident that higher values are achieved at 500 °C for almost all samples. The calorific values for some samples exceed the typical range of 15–22 MJ/kg [26], which is the average for bio-oil obtained from biomass, but remain significantly lower than the values for biodiesel, 37–39 MJ/kg [27], except for the light corn fraction sample Cn, condensed with acetone, which exceptionally had a calorific value of 43.60 MJ/kg.

The results of the elemental analysis are also provided in Table 1. The light fractions without acetone had the highest oxygen content, while the carbon content in the same fractions was the lowest. Additionally, it can be seen that the moisture content is also the highest in the LF for all biomass types, indicating that the oxygen present mainly originates from moisture. The low carbon content in the fractions without acetone suggests that most of the carbon is lost in the form of gases since there is no adsorbent that would retain carbon compounds. Therefore, acetone plays a significant role in removing water, which reduces the oxygen content in the samples and retains carbon in the form of liquid products that condense in the trap. It can also be concluded that the higher yield of the light fractions without acetone is, in fact, due to the higher moisture content, which gives “false” data showing that a higher yield of some bio-oil samples is obtained without acetone. The moisture content of the heavy fractions was below 10% in all samples, which may indicate that only some of the oxygen comes from moisture. However, a significant amount of oxygen is also found in organic compounds. The sulfur content (from 0.00 to 0.28%), determined by elemental analysis, is consistent and low.

The content of toxic elements, such as Pb, Cd, As, Cr, Hg, and Cu, was analyzed to assess whether these elements are present in the samples and to what extent. Although standards such as ASTM D7544 [28] and EN 16900 [29] do not define the maximum allowable concentrations of heavy metals in bio-oil, some studies have reported the presence of these elements in bio-oils. Based on the results obtained in this study, the concentrations of Pb, Cd, As, and Hg are generally lower or comparable to average values reported in the literature [30,31], while Cr and Cu concentrations are variable but still lower than the highest levels reported in previous studies [30,31,32]. Considering these findings and the concentrations measured in this study, it is recommended that bio-oils containing significant amounts of heavy metals should not be used as fuel to avoid potential emissions of toxic elements into the environment and adverse effects on living organisms. It was observed that at a pyrolysis temperature of 400 °C, the use of acetone resulted in a slightly higher retention of lead and copper in the liquid fraction compared to samples where no solvent was used. The highest amount of copper, 38.7 mg/kg, was recorded in the heavy tomato fraction. The highest chromium concentration, 65.67 mg/kg, was also recorded in the heavy tomato fraction, while chromium was detected in lower concentrations in the other heavy fractions. For most light fractions where acetone was used, as well as those where it was not, chromium concentrations were below 0.35 mg/kg, except for tomato’s LF-Ac at 400 °C, corn’s (Cn) LF at 500 °C and corn’s (Cz) LF at 500 °C, where chromium concentrations up to 3.46 mg/kg were recorded. The results show that the values for Cd and Hg were below the detection limit (<0.15 mg/kg) and values for As were below 0.25 mg/kg. The presence of other elements may be related to the soil in which the crops were grown, especially if the plants show an affinity for accumulating specific elements [33]. Other elements whose concentrations were determined were total S, Si, Ca, K, P, and Mg, which may affect the ash content [34]. Based on the results in Table 2, the Si, P, and Mg concentrations were below the detection limit (<100 mg/kg). A Ca concentration exceeding 100 mg/kg was detected only in the Tobacco HF sample at 500 °C (209.23 mg/kg). Potassium concentrations above 100 mg/kg were observed only in the heavy fractions of all biomass types. Notably, higher K concentrations were found at elevated temperatures in tomato and tobacco, while the opposite trend was observed in both maize species. Sulfur concentrations above 100 mg/kg were present in most samples. When comparing the light fractions where acetone was used with those where it was not used, it is evident that acetone facilitates the retention of compounds with S, resulting in higher S concentrations in these fractions. In addition, comparing the results at the two different temperatures reveals that most fractions show higher S concentrations at elevated temperatures. However, even when present, these elements do not significantly affect the ash content. This suggests that the elements are present in compounds that do not leave ash upon combustion. The absence of ash is important because increased ash content reduces the calorific value of bio-oil and, consequently, the efficiency of bio-oil as a fuel [35].

### 2.3. FTIR-Functional Group Analysis

**Figure 2:**
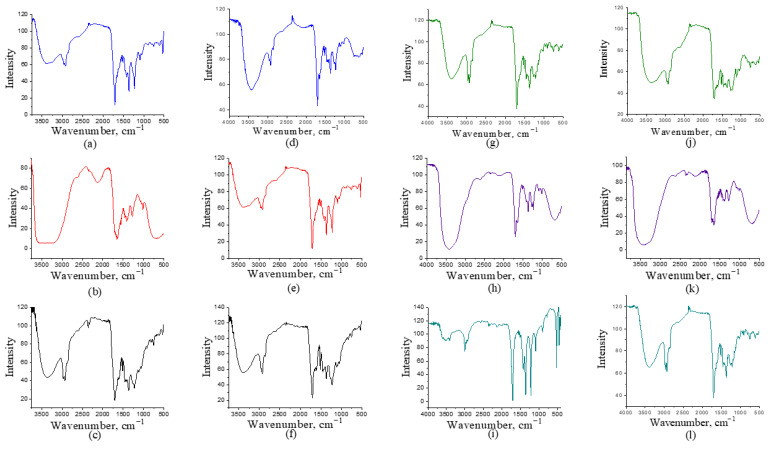
FTIR analysis of tomato at 400 °C: (**a**) light fraction with acetone; (**b**) light fraction without acetone; (**c**) heavy fraction; Tomato at 500 °C: (**d**) light fraction with acetone; (**e**) light fraction without acetone; (**f**) heavy fraction; Tobacco at 400 °C: (**g**) light fraction with acetone; (**h**) light fraction without acetone; (**i**) heavy fraction; Tobacco at 500 °C: (**j**) light fraction with acetone; (**k**) light fraction without acetone; (**l**) heavy fraction.

**Figure 3:**
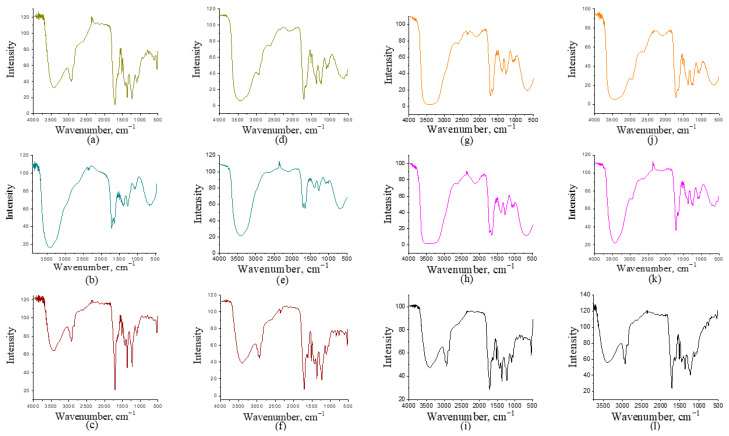
FTIR analysis of corn Cn at 400 °C: (**a**) light fraction with acetone; (**b**) light fraction without acetone; (**c**) heavy fraction; corn Cn at 500 °C: (**d**) light fraction with acetone; (**e**) light fraction without acetone; (**f**) heavy fraction; corn Cz at 400 °C: (**g**) light fraction with acetone; (**h**) light fraction without acetone; (**i**) heavy fraction; corn Cz at 500 °C: (**j**) light fraction with acetone; (**k**) light fraction without acetone; (**l**) heavy fraction.

The liquid pyrolysis fractions were analyzed using FTIR spectroscopy to identify functional groups present in the samples. The FTIR spectra are shown in Figure 2 and Figure 3. Differences are observed within the same type of biomass depending on the fraction analyzed and between samples obtained at different temperatures. The results of the FTIR analysis do not follow a clear pattern. A broad peak is observed at wavelengths between 3000 and 3500 cm^−1^, corresponding to vibrations of the O-H bond, indicating the presence of alcohols and phenols, which is expected, since pyrolysis of lignin typically produces phenols [36]. This peak was detected in all samples. At wavenumbers over 3500 cm^−1^, peak also correspond to O-H vibrations. This stretched peak often indicates the presence of water [36], which can be attributed to moisture in the samples. The highest moisture content is found in samples where acetone was not used, as shown in Figure 2b,e,h,k. In the case of corn, a stretched peak at the same wavelength is observed in both samples where acetone was used and those where it was not (Figure 3).

The stretching observed between 1650 and 1750 cm^−1^ corresponds to the C=O bond, indicating the presence of ketones, carboxylic acids, and aldehydes. The presence of the -C-H bond is confirmed by the stretching vibration peak between 1350 and 1480 cm^−1^. Absorption in the 1210 to 1320 cm^−1^ is associated with the functional group C-O, which may originate from acids, while absorption from alcohols and phenols is observed between 970 and 1250 cm^−1^. The absorption peak corresponding to the aromatic C=C bond is between 1500 and 1600 cm^−1^, while the fingerprint region is below 1500 cm^−1^. In the range of 2800 to 3000 cm^−1^, absorption occurs due to C-H bonds in saturated and unsaturated aliphatic and aromatic compounds [37,38,39,40].

Tobacco samples (Figure 2g–l) show more pronounced peaks in the aromatic region (1500–1600 cm^−1^), suggesting a higher abundance of aromatic compounds compared to tomato samples. Tomato samples (Figure 2a–f), on the other hand, display more intense carbonyl bands around 1700 cm^−1^, indicating a greater presence of carbonyl compounds. When comparing the effect of temperature on tomato and tobacco samples (Figure 2), it can be observed that higher temperature (500 °C) generally leads to more intense and sharper peaks in the aromatic region and a decrease in the intensity of the –OH band. This suggests dehydration and stronger formation of aromatic structures at elevated temperatures. For the light fractions obtained with acetone (Figure 2a,d,g,j), the carbonyl bands in the region around 1700 cm^−1^ and the C–O region are more prominent, showing that acetone favors the extraction of oxygenated compounds. In contrast, the light fractions without acetone (Figure 2b,e,h,k) exhibit weaker carbonyl peaks and more pronounced aliphatic C–H bands, indicating a somewhat simpler composition.

The spectra of heavy fractions (Figure 2c,f,i,l) show stronger and broader peaks in the low-frequency region (1200–700 cm^−1^), which may point to a higher content of more complex, high-polymeric, and aromatic structures, and consequently a lower content of light oxygenated components.

When comparing the light fractions obtained with acetone for the two types of corn (Figure 3a,d,g,j), a relatively stronger and better-defined carbonyl band around 1740–1700 cm^−1^ can be observed compared to the light fractions obtained without acetone (Figure 3b,e,h,k), which follows the same trend as observed for tomato and tobacco. The presence of the C=O vibration indicates a higher proportion of oxygenated, polar compounds such as ketones or esters. This is assumed to result from better condensation of these compounds in the presence of acetone, which, as a polar solvent, favors the retention and extraction of oxygenated compounds from pyrolysis vapors.

The presence of esters and alcohols is further confirmed by the fact that the light fractions with acetone show stronger C–O peaks in the range of 1300–1000 cm^−1^. In contrast, the light fractions without acetone exhibit more intense aliphatic C–H stretching peaks (~2950–2850 cm^−1^) and weaker carbonyl bands, indicating a simpler and less polar composition. Regarding the effect of temperature on the FTIR spectra, it can be concluded that at higher temperatures the –OH band becomes narrower, while aromatic C=C absorptions (around 1600 cm^−1^) become more intense, indicating dehydration and aromatization of components at elevated temperatures, as was also the case for tomato and tobacco. At 500 °C, the spectra of light fractions with acetone (Figure 3d,j) sometimes show sharper and more defined carbonyl bands and stronger fingerprint features compared to their 400 °C counterparts (Figure 3a,g). This suggests that higher temperatures favor the formation of more stable oxygenated monomeric aromatics and low-molecular-weight ketones/esters, while also promoting more intense thermal degradation of biopolymers into more aromatic products. In the heavy fractions at higher temperatures (Figure 3f,l), even more pronounced and complex signals were observed, which is consistent with the formation of higher-molecular-weight compounds. When comparing the two corn types (Figure 3a–f,g–l), the differences are subtle but consistent. The FTIR spectra for Cz (Figure 3g–l) show somewhat stronger aromatic bands in the region of 1600–1500 cm^−1^ and a richer fingerprint below 1400 cm^−1^ compared to Cn (Figure 3a–f), which may indicate differences in composite composition, i.e., a different cellulose/hemicellulose to lignin ratio, leading to the formation of different products under the same pyrolytic conditions. Conversely, some Cn spectra (particularly light fractions at lower temperature) retain a more pronounced –OH band and relatively stronger aliphatic C–H features, suggesting a higher content of light oxygenated and aliphatic compounds in these samples.

### 2.4. GC-MS—Chemical Composition Analysis

**Figure 4:**
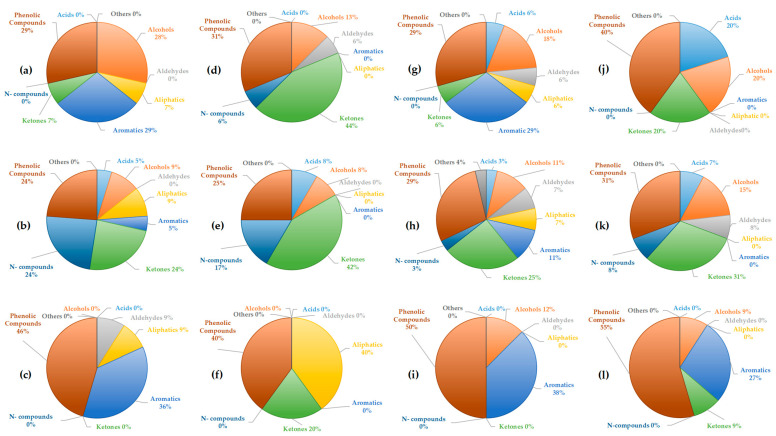
Share of acids, alcohols, aldehydes, aliphatics, aromatics, ketones, nitrogen, phenolic, and other compounds obtained by GC-MS analysis of liquid fractions produced by pyrolysis of tomato at 400 °C: (**a**) light fraction with acetone; (**b**) light fraction without acetone; (**c**) heavy fraction; pyrolysis of tomato at 500 °C: (**d**) light fraction with acetone; (**e**) light fraction without acetone; (**f**) heavy fraction; pyrolysis of tobacco at 400 °C: (**g**) light fraction with acetone; (**h**) light fraction without acetone; (**i**) heavy fraction; pyrolysis of tobacco at 500 °C: (**j**) light fraction with acetone; (**k**) light fraction without acetone; (**l**) heavy fraction.

**Figure 5:**
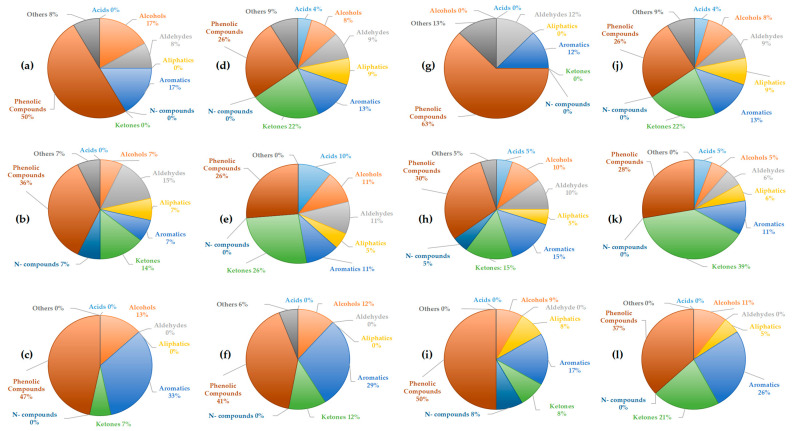
Share of acids, alcohols, aldehydes, aliphatics, aromatics, ketones, nitrogen, phenolic, and other compounds obtained by GC-MS analysis of liquid fractions produced by pyrolysis of corn Cn at 400 °C: (**a**) light fraction with acetone; (**b**) light fraction without acetone; (**c**) heavy fraction; pyrolysis of corn Cn at 500 °C: (**d**) light fraction with acetone; (**e**) light fraction without acetone; (**f**) heavy fraction; pyrolysis of corn Cz at 400 °C: (**g**) light fraction with acetone; (**h**) light fraction without acetone; (**i**) heavy fraction; pyrolysis of corn Cz at 500 °C: (**j**) light fraction with acetone; (**k**) light fraction without acetone; (**l**) heavy fraction.

The more detailed chemical composition of the liquid fractions was analyzed using GC-MS and compared with previous studies [41]. GC-MS analysis provides insights into the complexity of the obtained liquid fractions and the classes of compounds present [42]. The identified compounds in each fraction were categorized into the following classes: acids, phenolic compounds, ketones, aldehydes, aromatic compounds, aliphatic compounds, nitrogen compounds, and other compounds not classified within these categories. The analysis performed was qualitative. The proportions of each compound class in the obtained fractions at two temperatures, 400 °C and 500 °C, with and without using acetone, are presented in Figure 4 and Figure 5. GC-MS analysis performed after acetone evaporation confirmed that traces of acetone were not present in the bio-oil.

In the light fractions of tomato biomass obtained with acetone (Figure 4a,d), phenolic compounds were dominant (29–31%), followed by aromatics (29%) at 400 °C. At the higher temperature, ketones became more prominent (44%), accompanied by an increase in N-compounds (6%), while aromatic compounds disappeared. This trend suggests that higher temperatures favor thermal decomposition toward ketonic structures. In the light fractions without acetone (Figure 4b,e), N-compounds were more pronounced (17–24%), together with ketones (24–42%), while aromatics and alcohols were present in lower proportions. This observation indicates that acetone may favor the retention and extraction of more polar oxygenated and nitrogenous compounds. Also, this supports literature findings that higher temperatures favor the formation of oxygenated ketonic structures from cellulose, while the share of aromatics decreases [43]. In the heavy fractions (Figure 4c,f), phenolic compounds were predominant (46–40%), accompanied by a high content of aromatics (36%) at 400 °C and aliphatics (40%) at 500 °C. The absence of ketones suggests that the heavy fraction contains more stable aromatic/phenolic structures, which is consistent with the general pyrolytic behavior of lignin [44]. Moreover, the increase in aliphatics at higher temperature indicates the occurrence of secondary reactions leading to the degradation of primary compounds.

For tobacco biomass, the light fractions with acetone (Figure 4g,j) at 400 °C showed a balanced distribution of aromatics and phenolics (29% each), along with ketones (6%). At the higher temperature, phenolic compounds became dominant (40%), accompanied by ketones and acids (20%). The higher presence of oxygenated compounds at higher temperatures is in contrast to the tomato biomass. In the light fractions without acetone (Figure 4h,k), ketones (25–31%) and nitrogen-containing compounds (3–8%) were more abundant, whereas aromatics and phenolics were present to a moderate extent. Similar to the case of tomato, this indicates that more polar components are better represented in the absence of acetone. In the heavy fractions of tobacco, aromatics (50–55%) and phenolics (38%) were dominant, while ketones were present only in minor amounts.

These findings are consistent with the results obtained for tomato heavy fractions. A comparison of these two biomasses suggests that the pyrolysis of tomato results in a higher proportion of ketones in the light fractions, particularly at 500 °C, while tobacco yields more phenolic compounds and acids. Increasing temperature generally leads to higher proportions of phenolics and ketones, but also favors the formation of acids in tobacco. This trend aligns with literature showing that extended residence time and oxidative reactions during tobacco pyrolysis lead to the accumulation of acetic and other short-chain acids [45]. Aromatic compounds tend to decrease in tomato fractions, while in tobacco they are retained in the heavy fractions, confirming the stability of lignin-derived compounds in this phase of the bio-oil [44]. Overall, light fractions without acetone exhibit higher contents of ketones and nitrogenous compounds, whereas the heavy fractions are dominated by phenolic and aromatic compounds, with negligible amounts of lighter oxygenated fractions.

In the light fractions of corn Cn obtained with acetone (Figure 5a,d), phenolic compounds were dominant (26–50%), followed by aromatics (13–17%) and, to a lesser extent, ketones (0–22%). As observed, the proportion of ketones increased with higher temperature, while phenolics decreased, suggesting that higher temperatures promote the formation of lighter oxygenated compounds (ketones) at the expense of phenolic structures. In the fractions without acetone (Figure 5b,e), a broader distribution was observed, including phenolics (26–36%), aldehydes (11–15%), and ketones (14–26%), with minor contributions from aliphatic and aromatic compounds. It can be noted that in the absence of acetone, aldehydes and ketones became more prominent. In the heavy fractions (Figure 5c,f), phenolics (41–47%) and aromatics (29–33%) were predominant, with only a small proportion of ketones (7–12%). This indicates a trend toward the stabilization of phenolic and aromatic components in corn. In the pyrolysis of corn Cz, the light fraction with acetone (Figure 5g,j) was highly enriched in phenolics, with 63% at 400 °C and 26% at 500 °C. At the higher temperature, a more even distribution was observed among different compound groups: aromatics (13%), ketones (22%), aldehydes (9%), along with smaller amounts of alcohols and aliphatics. This suggests that higher temperatures promote decomposition and secondary reactions, leading to the breakdown of phenolic structures and the formation of a broader spectrum of compounds. In the light fraction without acetone (Figure 5h,k), ketones (15–39%) were dominant, followed by phenolics (28–30%) and aromatics (11–15%). Here again, the pronounced presence of ketones indicates a greater extraction of polar oxygenated products in the absence of acetone. In the heavy fraction of Cz (Figure 5i,l), phenolics (37–50%) and aromatics (17–26%) were predominant, with a smaller contribution of ketones (8–21%). This further confirms the stability of phenolic–aromatic structures in the heavy fraction.

A comparison of the two corn types reveals that Cn exhibited moderately distributed fractions with significant proportions of aldehydes and ketones, whereas Cz showed an extremely phenol-dominant profile at 400 °C (63%), shifting toward a broader distribution with higher ketone and aromatic contents at 500 °C. Thus, Cz was richer in phenolic compounds, while Cn produced more diverse products. Temperature was found to increase the proportion of ketones and aldehydes in both corn types at higher temperatures, while reducing the phenolic fraction, except in heavy fractions where phenolics remained dominant. Regarding the effect of acetone on light fractions, it can be concluded that fractions without solvent displayed higher proportions of ketones and aldehydes, suggesting that acetone retains more polar fractions. In heavy fractions, phenolic and aromatic compounds remained dominant, highlighting the stability of these structures [44]. Overall, the pyrolysis of corn clearly demonstrated fraction differentiation: heavy fractions were phenolic–aromatic in nature, while the composition of light fractions depended on acetone—phenolics dominated with acetone, whereas ketones and aldehydes were more prevalent without acetone.

Summary, GC–MS analysis of liquid fractions obtained from the pyrolysis of tomato, tobacco, and corn at 400 and 500 °C revealed significant differences depending on the biomass, temperature, and type of fraction (light fraction with or without acetone, heavy fraction). In general, heavy fractions were dominated by phenolic and aromatic compounds, while light fractions contained mainly ketones, aldehydes, and acids. This is consistent with literature reports indicating that lignin pyrolysis yields phenols and aromatics, whereas cellulose and hemicellulose mainly produce furans, aldehydes, ketones, and acids [43,44]. Acetone does not exhibit a consistent trend in influencing the proportions of individual compound classes, except that in most cases, it favors the production of bio-oil with a smaller number of compound classes and a higher proportion of phenolic compounds. In the heavy fraction, compounds with higher molecular mass are dominant, which was expected, while the light fraction contains a greater or approximately equal number of compounds with lower molecular mass compared to heavier compounds. Acetone did not affect the diversity of compounds but rather its affinity for specific types, resulting in a lower number of identified compounds in almost all fractions. The use of acetone favored phenolic, alcoholic, aromatic compounds, and ketones in tomatoes and tobacco, while in corn, phenolic, aromatic, alcoholic compounds, and aldehydes were predominant which is in agreement with literature data on the solvent effect in bio-oil extraction [46]. Overall, the results confirm that higher temperatures promote the formation of ketones and acids, while phenolic dominance remains stable in heavy fractions, confirming that the feedstock’s chemical composition plays a crucial role in product distribution during pyrolysis [47].

Additionally, it can be concluded from Table S1 that phenol and methoxy-phenol are the compounds that are present in all obtained fractions.

## 3. Materials and Methods

**Figure 6:**
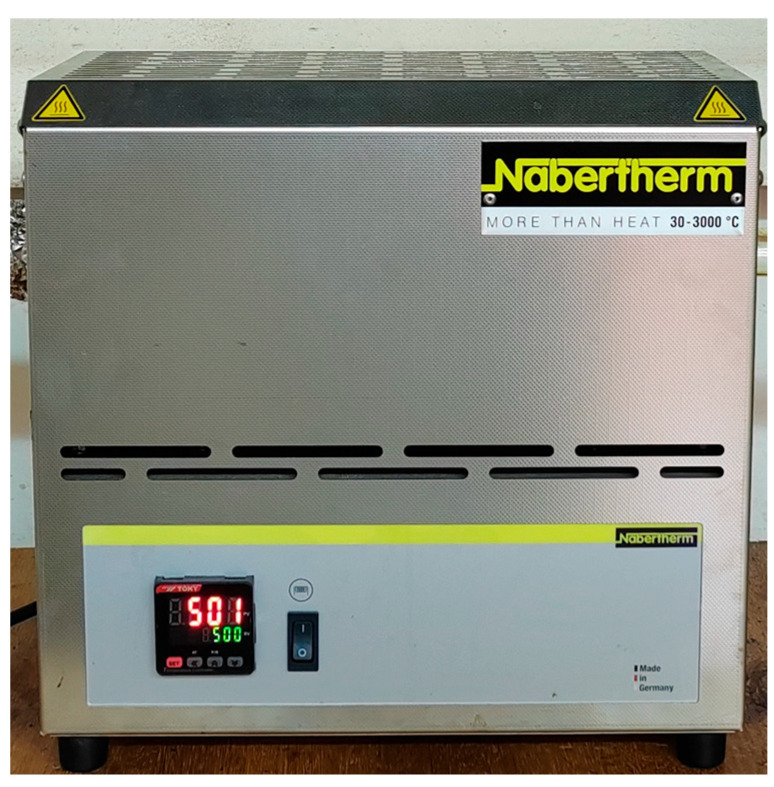
Nabertherm tube furnace (RD 30/200/11, Nabertherm, Lilenthal, Germany).

Waste biomass samples of tobacco, tomato, and two types of corn stalks (designated as Cn and Cz) were prepared for pyrolysis by air drying until the moisture content dropped below 10% and then ground to a particle size less than 5 mm. The content of lignin, cellulose, and hemicellulose was determined using the procedure described in detail by Isailović et al. (2024) and Vukićević et al. (2024), while thermogravimetric analysis (TGA) of the initial biomass was also performed [41,48]. Pyrolysis was conducted at two different temperatures, 400 and 500 °C, to compare the yield and chemical composition of the obtained liquid fractions. A Nabertherm tube furnace (RD 30/200/11, Nabertherm, Lilenthal, Germany) was used for pyrolysis (Figure 6), which has a higher capacity than the Carbolite furnace (MTF 10/15/130, Carbolite, Hope, Derbyshire, UK) used in our previous studies [41,48]. Consequently, more significant amounts of sample were pyrolyzed in the Nabertherm furnace, from about 3 to 5 g. The samples were placed in a preheated furnace and subjected to pyrolysis for 30 min at the specified temperature. The carrier gas was nitrogen, with a 150 mL/min flow rate.

For each biomass sample and each given temperature, pyrolysis was first performed without solvent in the recipient trap and then with acetone in the recipient trap. This way, three liquid fractions were collected for each biomass sample and each temperature. The light liquid fraction without acetone (LF) was collected in a recipient trap of known mass. After pyrolysis, the collected liquid fraction was weighed together with the trap. The light liquid fraction with solvent (LF-Ac) was collected in a recipient trap containing 20 mL of acetone, where the acetone was evaporated in a nitrogen stream after pyrolysis. The amount of the liquid fraction with acetone (LF-Ac) was determined again from the mass differences. The heavy fraction (HF) represented the products with higher boiling points, which remained in the tube and did not reach the recipient trap. This fraction was collected by rinsing the tube and the solid residue remaining inside the tube with 20 mL of acetone, after which the acetone was evaporated in a nitrogen stream. The proportion of the heavy fraction represented the sum of the heavy fractions obtained in experiments with and without using acetone to collect the light fraction, expressed relative to the total amount of initial biomass. The bio-oil yield related to each specific fraction was determined based on the initial amount of biomass, according to the following equation:

ω = m*_a_* − m*_b_*/m*_s_* × 100%, (1)

ω is the share of the liquid fraction; m*_a_* is the mass of the filled receiving vessel after pyrolysis; m*_b_* is the mass of the empty receiving vessel before pyrolysis; m*_s_* is the weighed mass of the biomass sample.

All obtained fractions were characterized by physicochemical parameters that included moisture content using the standard “Petroleum products-Determination of water-Coulometric Karl Fischer titration method” [49] on an 831 KF Coulometer (Metrohm, Herisau, Switzerland)), ash content [50], pH [51], elemental analysis (on a CHNS Elementar-Vario Macro Cube (Elementar, Langenselbold, Germany) for carbon, hydrogen, nitrogen, and sulfur analysis [52,53,54] (the oxygen content was calculated as a difference of 100%), and calorific value (on an instrument IKA C400 (IKA, Staufen, Germany) [55]. FTIR analysis for liquid bio-oil samples (~0.01 cm^3^) were conducted on an IRAffinity-1 FTIR spectrophotometer (Shimadzu, Kyoto, Japan), where samples were placed between two KBr plates. Spectra were obtained using a transmission technique. The infrared spectra were in the wavelength range 4000–400 cm^−1^ and the measurement resolution was 4 cm^−1^. ICP-OES analysis was performed using the ICP-OES technique (“Spectroblue TI”-Spectro, Kleve, Germany) following the standard method EPA 200.7 [56], and GC-MS analysis was conducted on the Varian 450-GC with 220-MS, Varian, Memphis, TN, USA. All procedures are detailed described by Isailović et al. (2024) [41].

## 4. Conclusions

This study presents a systematic analysis of the pyrolysis of four types of biomass—tomato, tobacco, and two maize varieties—conducted at 400 and 500 °C, with and without the use of an organic solvent. Both light and heavy fractions were collected. The results indicate a positive effect of acetone on the liquid yield for both maize varieties, while a negative effect was observed for tobacco and tomato. The presence of the solvent also increased the pH value and significantly reduced the moisture content, as confirmed by elemental analysis (reduced oxygen content) and FTIR analysis, which revealed notable spectral differences. Furthermore, acetone improved the calorific value in both maize types, whereas the effect was minimal in tobacco and negligible in tomato. These findings suggest a potential advantage for biofuel production. GC-MS analysis revealed a significant proportion of phenolic compounds in all fractions, with phenol and methoxy-phenol being the only compounds consistently identified across all samples. Notably, in most cases, acetone favored the formation of fewer compound classes while increasing the yield of phenolic compounds. Depending on the intended application, this property can be exploited to produce bio-oils enriched in targeted compound classes.

The research results indicate that, from the aspect of pyrolytic degradation efficiency, the approach using an organic solvent proved to be more efficient. However, the solvent-free method also demonstrated satisfactory performance. This study contributes to clarifying the necessity of using organic solvents in such processes and supports further development of optimized solvent-free methods and underlines the strategic potential of both approaches, depending on the target products and sustainability priorities. Although acetone is an efficient and relatively acceptable solvent compared to other organic solvents, future research should be directed towards the use of solvents that combine high efficiency, low toxicity, good renewability, and biodegradability. The focus should be on optimizing solvent-free pyrolytic methods for various types of waste materials and using green chemistry and “green” solvents, such as water, bio-based derivatives, or, for example, supercritical CO_2_.

## Figures and Tables

**Table 1:** Physicochemical characterization of different bio-oil fractions (pH, moisture content, ash content, calorific value and elemental analysis) obtained at 400 and 500 °C.

Sample Name/Parameter		pH	Moisture Content, *w*/*w*, %	Ash Content, *w*/*w*, %	Calorific Value, MJ/kg	Elemental Analysis, *w*/*w*, %				
						N	C	H	S	O
Tomato LF-Ac	400 °C	4.30	5.78	0.00	26.60	0.84	44.47	8.81	0.00	45.88
	500 °C	5.05	26.32	0.00	27.49	2.92	43.68	9.96	0.06	43.38
Tomato LF	400 °C	4.52	79.45	0.00	27.49	1.45	10.74	9.29	0.00	78.52
	500 °C	4.99	77.50	0.01	26.34	1.68	19.84	11.63	0.01	66.85
Tomato HF	400 °C	5.69	2.76	0.07	24.96	2.14	56.77	9.08	0.00	32.01
	500 °C	6.06	6.67	0.17	26.22	6.34	66.56	11.36	0.02	15.72
Tobacco LF-Ac	400 °C	4.43	20.20	0.00	20.22	0.82	44.04	8.75	0.00	46.39
	500 °C	4.99	23.89	0.00	21.07	4.11	49.93	9.73	0.06	36.18
Tobacco LF	400 °C	3.75	51.69	0.00	19.44	0.79	26.69	9.88	0.00	62.64
	500 °C	3.88	57.25	0.05	14.70	0.16	10.05	1.41	0.00	88.38
Tobacco HF	400 °C	4.66	13.01	0.00	15.21	0.87	50.51	8.15	0.00	40.47
	500 °C	4.84	8.86	0.00	23.22	4.90	52.49	8.87	0.07	33.66
Cn LF-Ac	400 °C	4.01	7.10	0.03	24.85	0.80	40.70	5.46	0.28	52.76
	500 °C	4.08	12.83	0.00	43.60	0.92	43.94	7.97	0.07	47.10
Cn LF	400 °C	2.78	60.72	0.00	16.05	0.54	22.00	8.63	0.05	68.78
	500 °C	2.97	61.82	0.00	17.93	0.10	9.93	3.41	0.06	86.50
Cn HF	400 °C	3.42	7.96	0.04	26.07	1.91	50.44	7.27	0.06	40.32
	500 °C	3.59	8.05	0.07	27.23	0.01	12.42	1.91	0.13	85.51
Cz LF-Ac	400 °C	3.88	13.81	0.00	22.70	0.69	47.46	8.67	0.00	43.18
	500 °C	3.97	18.93	0.06	26.63	1.00	29.33	5.21	0.04	64.42
Cz LF	400 °C	3.16	65.48	0.00	21.98	1.06	32.69	8.39	0.02	57.84
	500 °C	3.24	67.34	0.03	23.67	0.35	18.17	10.70	0.03	70.75
Cz HF	400 °C	3.72	6.96	0.04	27.64	0.84	49.04	8.08	0.00	42.04
	500 °C	3.95	8.15	0.00	27.60	0.99	53.83	7.92	0.00	37.26

LF—light fraction condensed without acetone; LF-Ac—light fraction condensed with acetone; HF—heavy fraction.

**Table 2:** Toxic elements content (Pb, Cd, As, Cr, Hg, and Cu), and content of total S, Si, Ca, K, P, and Mg in mg/kg.

Sample Name/Metal Content, mg/kg		Pb	Cd	As	Cr	Hg	Cu	S_total_	Si	Ca	K	P	Mg
Tomato LF-Ac	400 °C	0.26	<0.15	<0.25	2.55	<0.15	1.51	429.7	<100	<100	<100	<100	<100
	500 °C	0.31	<0.15	<0.25	<0.35	<0.15	0.31	810.8	<100	<100	<100	<100	<100
Tomato LF	400 °C	0.11	<0.15	<0.25	<0.35	<0.15	1.24	157.3	<100	<100	<100	<100	<100
	500 °C	0.20	<0.15	<0.25	<0.35	<0.15	<0.30	120.3	<100	<100	<100	<100	<100
Tomato HF	400 °C	0.10	<0.15	<0.25	<0.35	<0.15	1.14	111.5	<100	<100	853.4	<100	<100
	500 °C	0.51	0.28	<0.25	65.67	<0.15	38.37	786.2	<100	<100	1227	<100	<100
Tobacco LF-Ac	400 °C	0.41	<0.15	<0.25	<0.35	<0.15	<0.30	344.6	<100	<100	<100	<100	<100
	500 °C	0.26	<0.15	<0.25	<0.35	<0.15	1.01	515.2	<100	<100	<100	<100	<100
Tobacco LF	400 °C	0.01	<0.15	<0.25	<0.35	<0.15	<0.30	106.4	<100	<100	<100	<100	<100
	500 °C	0.45	<0.15	<0.25	<0.35	<0.15	<0.30	124.2	<100	<100	<100	<100	<100
Tobacco HF	400 °C	0.09	<0.15	<0.25	3.39	<0.15	1.34	<100	<100	<100	520.5	<100	<100
	500 °C	0.30	<0.15	<0.25	4.33	<0.15	2.77	707.2	<100	209.23	3485.0	<100	<100
Cn LF-Ac	400 °C	0.38	<0.15	<0.25	<0.35	<0.15	0.88	367.1	<100	<100	<100	<100	<100
	500 °C	0.22	<0.15	<0.25	<0.35	<0.15	<0.30	657.4	<100	<100	<100	<100	<100
Cn LF	400 °C	0.19	<0.15	<0.25	<0.35	<0.15	<0.30	311.1	<100	<100	<100	<100	<100
	500 °C	0.44	<0.15	<0.25	0.74	<0.15	0.65	112.8	<100	<100	<100	<100	<100
Cn HF	400 °C	0.16	<0.15	<0.25	5.06	<0.15	1.94	<100	<100	<100	332.1	<100	<100
	500 °C	0.16	<0.15	<0.25	<0.35	<0.15	<0.30	<100	<100	<100	230.0	<100	<100
Cz LF-Ac	400 °C	0.24	<0.15	<0.25	<0.35	<0.15	<0.30	390.4	<100	<100	<100	<100	<100
	500 °C	0.17	<0.15	<0.25	<0.35	<0.15	<0.30	680.7	<100	<100	<100	<100	<100
Cz LF	400 °C	0.19	<0.15	<0.25	<0.35	<0.15	<0.30	236.6	<100	<100	<100	<100	<100
	500 °C	0.25	<0.15	<0.25	3.46	<0.15	<0.30	348.7	<100	<100	<100	<100	<100
Cz HF	400 °C	0.54	<0.15	<0.25	3.23	<0.15	1.75	141.0	<100	<100	489.8	<100	<100
	500 °C	0.35	<0.15	<0.25	13.79	<0.15	<0.30	<100	<100	<100	441.6	<100	<100

## Data Availability

The original contributions presented in this study are included in the article/Supplementary Materials. Further inquiries can be directed to the corresponding author.

## Supplementary Materials

The following supporting information can be downloaded at: https://www.mdpi.com/article/10.3390/molecules30193945/s1, Table S1: GC-MS analysis of liquid fractions-list of determined compounds.
