# Supplementary material for: Solvent-Assisted Vapor Condensation: A Strategy to Enhance Bio-Oil Yield and Quality from the Pyrolysis of Agro-Industrial Waste
Source: Molecules. 2025 Oct 1;30(19):3945. doi: 10.3390/molecules30193945 (PMC12525771; doi:10.3390/molecules30193945)
Supplement: Supplementary file 1 [file molecules-30-03945-s001.zip › molecules-3884818-supplementary.pdf]

Table S1. GC-MS analysis of liquid fractions - list of determined compounds

Compounds	Tomato						Tobacco						Cn						Cz					
	LF-Ac		LF		HF		LF-Ac		LF		HF		LF-Ac		LF		HF		LF-Ac		LF		HF	
	I	II	I	II	I	II	I	II	I	II	I	II	I	II	I	II	I	II	I	II	I	II	I	II
Methyl glycolate	-	-	+	-	-	-	+	+	+	+	-	-	-	+	-	+	-	-	-	+	+	+	-	-
Pyrazine	-	-	+	-	-	-	-	-	-	-	-	-	-	-	-	-	-	-	-	-	-	-	-	-
Pyridine	-	-	+	-	-	-	-	-	-	-	-	-	-	-	-	-	-	-	-	-	-	-	-	-
Pentanone	-	-	+	-	-	-	-	-	-	+	-	-	-	-	+	-	-	-	-	-	+	+	-	-
Branded octane	-	-	-	-	-	-	-	-	-	-	-	-	-	+	-	-	-	-	-	+	-	-	-	-
Pentamethylheptane	-	-	-	-	-	-	-	-	+	-	-	-	-	-	-	+	-	-	-	-	-	-	-	-
Pentanal	-	-	-	-	-	-	-	-	-	-	-	-	-	-	+	+	-	-	-	-	+	-	-	-
Dimethylpiperidine	-	-	+	+	-	-	-	-	-	+	-	-	-	-	-	-	-	-	-	-	-	-	-	-
Cyclopentanone	-	+	-	-	-	-	-	-	+	-	-	-	-	+	-	-	-	-	-	+	-	+	-	-
Methylentenone	-	+	-	+	-	+	-	-	-	-	-	-	-	-	-	-	-	-	-	-	-	-	-	-
Dimethylpentenoic acid	-	-	-	+	-	-	-	-	-	-	-	-	-	-	-	-	-	-	-	-	-	-	-	-
Methylpyridine	-	+	-	-	-	-	-	-	-	-	-	-	-	-	-	-	-	-	-	-	-	-	-	-
Furancarboxaldehyde	-	-	-	-	-	-	+	-	+	-	-	-	+	+	+	-	-	-	+	+	-	-	-	-
Furaldehyde	-	-	-	-	-	-	-	-	-	-	-	-	-	-	-	-	-	-	-	-	-	-	-	-
Methyl-1H-pyrazole	-	-	+	-	-	-	-	-	+	-	-	-	-	-	-	-	-	-	-	-	+	-	-	-
Octanol	+	-	-	-	-	-	+	+	-	+	-	-	+	-	-	-	+	-	-	-	-	-	-	-
Furanmethanol	+	+	+	+	-	-	+	-	+	+	-	-	-	+	-	-	-	+	-	+	+	-	-	+
Ethylbenzene	-	-	-	-	-	-	-	-	-	-	-	-	-	-	-	-	+	+	-	-	-	-	-	+
Methylcyclopenten-one	+	+	+	+	-	-	+	+	+	+	-	-	-	+	-	+	-	-	-	+	+	+	-	+
Dimethylhexadiene	-	-	+	-	-	-	+	-	-	-	-	-	-	-	+	-	-	-	-	-	+	-	-	-
Tetramethylcyclobutene	-	-	+	-	-	-	-	-	+	-	-	-	-	-	-	-	-	-	-	-	-	+	-	-
Alpha, beta-crotonolactone	-	-	-	-	-	-	-	-	+	-	-	-	-	+	+	-	-	-	-	+	+	-	-	-
Hexanediol	+	-	-	-	-	-	-	-	-	-	-	-	-	-	-	-	-	-	-	-	-	-	-	-
Methylfurancarboxaldehyde	-	-	-	-	-	-	-	-	+	-	-	-	-	+	-	+	-	-	-	+	-	+	-	-
Methylcyclopenten-one	-	+	+	-	-	-	-	-	+	-	-	-	-	+	-	+	-	-	-	+	-	+	-	+
Hexadienal	-	+	-	-	-	-	-	-	-	+	-	-	-	-	-	-	-	-	-	-	+	-	-	-
Phenol	+	+	+	+	+	+	+	+	+	+	+	+	+	+	+	+	+	+	+	+	+	+	+	+
Aniline	-	-	-	-	-	-	-	-	-	-	-	-	-	-	+	-	-	-	-	-	-	-	+	-
Decen-ol	-	-	-	-	-	-	-	-	+	-	-	-	-	-	-	-	-	-	-	-	-	-	-	-
Dimethylaminopyridine	-	-	-	+	-	-	-	-	-	-	-	-	-	-	-	-	-	-	-	-	-	-	-	-
Hydroxymethylcyclopenten-one	-	+	+	+	-	-	-	-	+	+	-	-	-	+	+	+	+	+	-	+	+	+	+	+
Dimethylcyclopenten-one	-	+	+	+	-	-	-	-	+	+	-	+	-	+	-	+	-	+	-	+	-	+	-	+
Methylphenol	-	+	+	-	+	-	+	-	+	-	-	+	-	+	-	+	+	+	-	+	+	+	+	+
Benzenmethanol	+	-	-	-	+	-	+	-	+	-	-	+	-	+	-	+	+	+	-	+	+	+	+	+
Methylbenzaldehyde	-	-	-	-	+	-	-	-	-	-	-	-	-	-	-	-	-	-	-	-	-	-	-	-
Methoxyphenol	+	+	+	+	+	+	+	+	+	+	+	+	+	+	+	+	+	+	+	+	+	+	+	+
Dimethylcyclohexen-one	-	-	-	-	-	-	-	-	+	-	-	-	-	-	-	-	-	-	-	-	-	-	-	-
(1H)-pyridinone	-	-	+	-	-	-	-	-	-	-	-	-	-	-	-	-	-	-	-	-	-	-	-	-
Ethylhydroxycyclopenten-one	-	-	-	-	-	-	-	-	+	-	-	-	-	-	-	+	-	-	-	-	-	+	-	-
Tetramethylpiperidine-one	-	+	-	+	-	-	-	-	-	-	-	-	-	-	-	-	-	-	-	-	-	-	-	-
Trimethylheptadien-one	-	-	-	-	-	+	-	-	-	-	-	-	-	-	-	-	-	-	-	-	-	-	-	-
Dimethylphenol	-	-	-	-	-	-	-	-	+	-	-	+	-	+	-	-	+	+	-	+	-	-	+	+
Ethylphenol	-	-	-	-	-	-	-	-	+	-	-	+	+	+	+	+	+	+	+	+	+	+	+	+
Hydroxymethylanisol	+	+	+	-	-	-	+	-	+	-	+	+	+	+	+	+	+	+	-	+	+	+	+	+
Benzendiol	-	-	-	-	-	-	-	-	-	-	-	-	-	-	-	+	-	-	-	-	-	-	-	-
Dihydrobenzofuran	-	-	-	-	-	-	-	-	-	-	-	-	+	+	-	-	-	+	+	+	-	-	-	-
Methoxybenzeneethanol	-	-	-	-	+	-	+	-	+	-	+	-	-	-	-	+	-	-	-	-	+	-	+	+
Ethylguaiacol	+	-	-	-	-	-	+	-	-	-	+	+	+	+	-	-	+	+	-	+	-	+	-	+
Methoxyvinylphenol	+	-	-	-	+	-	+	-	-	-	+	+	-	+	-	-	-	+	-	+	-	-	-	+
Hydroxymethylacetophenone	-	-	-	-	-	-	-	-	-	-	-	-	+	-	-	-	-	-	+	-	-	-	-	-
Dimethoxyphenol	+	+	+	-	+	-	+	-	+	+	+	+	+	+	+	+	+	+	-	+	+	+	+	+
Hydroxymethoxybenzoic acid	-	-	-	-	-	-	-	-	-	-	-	-	-	-	-	+	-	-	-	-	-	-	-	-
Trimethoxybenzene	-	-	-	-	-	-	-	-	-	-	-	-	-	-	-	-	+	+	-	-	-	-	-	-
Methoxypropenylphenol	-	-	-	-	-	-	+	-	+	-	+	-	-	-	-	-	-	+	-	-	-	-	-	+
Di-tert-butylphenol	+	+	+	+	+	-	-	-	+	+	-	-	+	-	+	-	+	-	+	-	+	-	-	-
Dodecene	-	-	-	-	-	-	-	-	-	-	-	-	-	+	-	-	-	-	-	+	-	-	+	+
Methyloctadecyne	+	-	-	-	+	+	-	-	-	-	-	-	-	-	-	-	-	-	-	-	-	-	-	-
Sulfonylbisbenzene	+	-	+	-	+	-	+	-	+	-	-	-	+	-	+	-	+	-	+	-	+	-	-	-

I - 400 ℃; II- 500 ℃; ′′+′′- compound identified;′′ - ′′-compound not identified
